# Apoptosis Activity of the Mouse Macrophage Cell Line J774A.1 Infected with a Recombinant BCG consisting the C-Terminus of Merozoite Surface Protein-1 of *Plasmodium falciparum*

**DOI:** 10.21315/tlsr2018.29.2.5

**Published:** 2018-07-06

**Authors:** Anis Fadhilah Zulkipli, Nor Munirah Zakaria, Mohamed Hussein Abdikarim, Maryam Azlan, Nurulasma Abdullah, Norazmi Mohd. Nor, Rapeah Suppian

**Affiliations:** School of Health Science, Universiti Sains Malaysia, Health Campus, 16150 Kubang Kerian, Kelantan, Malaysia

**Keywords:** Macrophage Apoptosis, Malaria, MSP-1C, Recombinant BCG, Apoptosis Makrofaj, Malaria, MSP-1C, BCG Rekombinan

## Abstract

Macrophage apoptosis exerts an efficient mechanism in controlling intracellular infection during innate immune response against various pathogens including malaria parasites. This study was carried out to determine the apoptosis activity in mouse macrophage cell line J774A.1 infected with a *Mycobacterium bovis* bacille Calmette-Guerin (BCG) clone and a recombinant BCG clone expressing the C-terminus of merozoite surface protein-1 (BCG-MSP1C) of *Plasmodium falciparum* for 48 h. In this study, a parent BCG cells was used as a control. The nuclear staining with Hoechst 33342 showed that the BCG-MSP1C cells was capable of increasing the nuclear condensation and morphological stages of apoptosis in the infected cells compared to the BCG-infected cells and the lipopolysaccharide (LPS)-stimulated cells. The flow cytometric analysis using Annexin-V and Propidium iodide (PI) staining confirmed that the BCG-MSP1C cells significantly increased the percentage of early apoptotic activity in the infected macrophage higher than the one stimulated by the parent BCG cells and LPS. This apoptotic response corresponded with the reduction of the anti-apoptotic Bcl-2 protein expression and higher p53 expression. The colorimetric assay demonstrated that the BCG cells capable of stimulating higher production of caspase-1, –3, –8 and –9 while the BCG-MSP1C cells stimulated the expression of caspase-1 and -9 in the infected macrophages, suggesting the involvement of mitochondrial-mediated (intrinsic) pathway of apoptosis. In conclusion, both the BCG and BCG-MSP1C cells are capable of inducing macrophage apoptosis activity in the mouse macrophage cell line J774A.1. This mechanism is important for the elimination of pathogens such as malaria parasite during the phagocytosis activity of macrophage. However, the BCG-MSP1C cells showed higher apoptosis activity than those produced by the parent BCG cells.

## Abstract (ms)

Apoptosis makrofaj merupakan mekanisme yang berkesan dalam mengawal jangkitan intrasel semasa gerak balas imun semulajadi terhadap pelbagai patogen termasuk parasit malaria. Kajian ini dijalankan untuk menentukan aktiviti apoptosis dalam sel makrofaj mencit J774A.1 yang dijangkiti klon *Mycobacterium bovis* BCG dan BCG rekombinan yang mengekspreskan terminus C protein permukaan merozoit-1 (BCG-MSP1C) daripada *Plasmodium falciparum* selama 48 jam. Kajian ini menggunakan sel BCG sebagai kawalan. Pewarnaan nukleus menggunakan Hoest 33342 menunjukkan bahawa sel BCG-MSP1C berupaya meningkatkan kondensasi nuklear dan peringkat morfologi apoptosis dalam sel makrofaj yang dijangkiti secara signifikan berbanding sel yang dijangkiti oleh sel BCG dan sel yang dirangsang dengan LPS. Analisis flow sitometri menggunakan pewarnaan Annexin-V dan PI membuktikan bahawa sel BCG-MSP1C meningkatkan peratusan aktiviti apoptotik awal didalam sel makrofaj mencit yang dijangkiti berbanding sel yang dijangkiti oleh BCG dan dirangsang dengan LPS. Gerak balas apoptosis yang ditunjukkan ini seiring dengan pengurangan pengekpresan protein anti-apoptotik Bcl-2 dan peningkatan pengekspresan protein p53. Ujian permeteran warna menunjukkan sel BCG berupaya meningkatkan mengekspreskan aktiviti kaspase-1, -3, -8 dan -9 manakala sel BCG-MSP1C hanya mengaktifkan pengekspresan kaspase-1 and -9 di dalam sel makrofaj yang dijangkiti, mencadangkan penglibatan laluan apoptosis mitokondria (intrinsik). Sebagai kesimpulan, kedua-dua sel BCG dan BCG-MSP1C berupaya meningkatkan aktiviti apoptosis di dalam sel makrofaj mencit, J774A.1. Mekanisme ini adalah penting untuk menyingkirkan patogen seperti parasit malaria semasa aktiviti fagositosis makrofaj. Walaubagaimanapun, sel BCG-MSP1C menunjukkan aktiviti apoptosis yang lebih tinggi berbanding sel BCG.

## INTRODUCTION

Malaria remains the public health concerns owing to the high rate of mortality and morbidity (Wiwanitkit 2011). It annually affects millions of people throughout the world, especially older people and pregnant ladies. Mostly, children under the age five years are vulnerable to life-threatening anaemia and cerebral malaria (World Health Organization 2015). The obligate intracellular parasite, *Plasmodium* is the causative agent of malaria disease. The infection is transmitted to humans through the saliva of the female *Anopheles* mosquitoes *(*Sinden & Gilles 2002*). Plasmodium falciparum* causes the most serious pathologies of malaria disease in human due to its capability to multiply rapidly in the blood. Infections with this parasite can be lethal in the absence of quick detection of the disease (Sinden & Gilles 2002*;* Snow *et al.* 2005; Ministry of Health Malaysia 2014; World Health Organization 2015).

The development of a safe and effective vaccine that elicits lasting immune responses against malaria has been a major agenda for controlling the disease due to the spread of drug-resistant parasites and insecticide-resistant mosquitoes in many parts of the world (Brogdon & McAllister 1998; Phillips 2001; Cravo *et al.* 2015). The clinical symptoms and pathologies associated with malaria occur during the blood stage infection. At this stage, the parasites express various antigens. Among these, the 19 kDa C-terminus of the merozoite surface protein-1 (MSP-1_19_) or also known as MSP-1C has been extensively studied as a blood-stage malaria vaccine candidate. A previous study showed that antibodies produced against the MSP-1C have been reported to be associated with protection from symptomatic malaria disease (Wan Omar *et al.* 2007).

*Mycobacterium bovis* bacilli Calmette-Guerin (BCG) is the only vaccine used for tuberculosis. It represents one of the most promising live vectors for the delivery of foreign antigen to the immune system, including malaria parasites (Bloom 1989). Previously, our group has constructed a recombinant BCG clone that consists the MSP-1C of *Plasmodium falciparum* (Nurul *et al.* 2010). Rigorous studies in mice have shown that our constructed vaccine represents a promising candidate to prevent malaria infection by inducing appropriate humoral and cellular immune responses.

The vaccine candidate is also capable of stimulating the production of a strong pro-inflammatory cytokines such as tumor necrosis factor (TNF-α), interleukin-1β (IL-1β) and nitric oxide (NO) and the expression of toll-like receptors in mouse macrophage cell line J774A.1 better than the parent BCG cells. Indeed, the previous finding had demonstrated that the phagocytic activity of macrophage infected with the BCG-MSP1C cells was increased, resulting in a significant reduction of macrophage viability as well as the viability of the BCG-MSP1C cells itself (Rapeah *et al.* 2010).

The recognition of mycobacteria such as BCG by macrophage involves the interaction of pattern recognition receptors such as toll like receptors (TLRs) with pathogen-associated molecular patterns (PAMPs) (Medzhitov & Janeway 2000; Akira & Takeda 2004; Akira *et al.* 2006). Signalling through TLRs may also activate a programmed cell death (apoptosis) response (Aliprantis *et al.* 2000) which is an important mechanism during the innate immune response. Apoptosis exerts protective activity against mycobacteria by preventing the release of intracellular components. Also, apoptosis prevents the dissemination of mycobacteria through sequestering the pathogens within apoptotic bodies leading to a reduction in bacterial viability (Behar *et al.* 2011).

Previous studies have shown that the pro-inflammatory response of macrophage induced by BCG was associated with apoptosis activity (Keane *et al*. 2000; Riendeau & Kornfeld 2003; Vanzembergh *et al*. 2011). However, information on the ability of the BCG-MSP1C cells to induce apoptosis activity in macrophage is still lacking and requires more study. Therefore, we hypothesized that this phenomenon involves macrophage apoptosis mechanism. To prove this hypothesis, this study was carried out to investigate the apoptosis activity in mouse macrophage cell line J774A.1 following the BCG and BCG-MSP1C infection. Macrophage apoptosis is an essential mechanism which exerts protective effects against mycobacteria during innate immune response (Schorey & Cooper 2003; Raja 2004; Briken & Miller 2008). We hope this study will provide a better understanding of the key immunological processes during a mycobacteria infection, and it helps in designing a safe and effective malaria vaccine.

## MATERIALS AND METHODS

### Preparation of Macrophage Culture

Mouse macrophage cell line J774A.1 (ATCC® TIB67™) was cultured in Dulbecco’s Modified Eagle’s Medium (DMEM) (Sigma, USA) supplemented with 1% of 100U/mL penicillin, 100 μg/ml streptomycin (Gibco, USA) and 10% fetal bovine serum (Gibco, USA). 5 × 105 cells/ml were grown in a 25 cm^2^ tissue culture flask and incubated at 37°C in the presence of 5% CO_2_ in a humidified incubator.

### *Mycobacterium* Culture

*Mycobacterium bovis* BCG was generously given by Professor Norazmi Mohd Nor from the School of Health Science, Universiti Sains Malaysia. The BCG-MSP1C containing the MSP-1C of *Plasmodium falciparum* was constructed by Nurul (2007) using assembly Polymerase chain reaction (PCR) in the previous study. The stability of the MSP-1C gene of *Plasmodium falciparum* in BCG-MSP1C cells was determined using PCR and the protein expression was determined by immunocytochemistry (ICC) analysis using specific antibody against MSP-1C (Dhaniah *et al*. 2014). The BCG and BCG-MSP1C cultures were grown on 7H11 agar supplemented with Oleic acid, albumin fraction V, dextrose and catalase (OADC) at 37°C. But, 15 μg/mL of kanamycin was added for BCG-MSP1C culture. After two weeks, a single colony of BCG and BCG-MSP1C from 7H11 agar was transferred into the 7H9 broth supplemented with OADC. 15 μg/mL of kanamycin was also added into the 7H9 broth for the BCG-MSP1C culture. The broth was incubated for one week (A_600_= ~0.8).

### Infection of Mouse Macrophage Cell Line J774A.1 with BCG and BCG-MSP-1C cells

After culturing for overnight, the mouse macrophage cell line J774A.1 (5 × 10^5^ cells/mL) were infected with the BCG or BCG-MSP1C cells (1 × 10^7^ cfu/ml) at a multiplicity of infection (MOI) of 1:20 in complete DMEM at 37°C in the presence of 5% CO_2_ for 48 h. Cells treated with LPS (100 ng/ml) were used as a positive control while untreated cells were used as a negative control.

### Detection of Cell Viability using Colorimetric MTT

Cell viability of infected and uninfected cells was performed in a 96 well flat bottom microplate using a colorimetric MTT (3-(4,5-dimethylthiazol-2-yl)-2,5-diphenyltetrazolium bromide) (Molecular Probes, USA) assay as described by the manufacturer.

### Detection of Apoptotic Cells using Hoechst 33342 Nuclear Staining Assay

In brief, the uninfected and infected cells were detached using a scraper and the detached cells were harvested by centrifugation at 125 × g for 5 min at room temperature. The pellet was washed twice with cold phosphate-buffered saline (PBS) (Amresco, USA) and dissolved in 1 mL PBS. The cell suspension was layered on poly-L-lysin slide (Thermo scientific, USA) and dried. Next, the slides were fixed with cold methanol (Merck, USA) for 15 min at room temperature. 0.2% of triton-x solution (Sigma, USA) was later added for 2 min. After that, the slides were rinsed three times with PBS and incubated with Hoechst stain (1 μg/mL) (Pierce Biotechnology, USA) for 30 min in a dark condition at room temperature. The slides were washed again for three times with PBS. The slides were examined using a fluorescence microscope to detect apoptosis.

### Detection of Apoptotic Cells using Flow Cytometer

The apoptotic cells were quantified using FITC Annexin-V Apoptosis Detection Kit II (BD, USA) using a flow cytometer according to manufacturer’s instructions. Briefly, the uninfected and infected cells were detached using a scraper and the detached cells were harvested by centrifugation at 125 × g for 5 min at room temperature. The cells pellet was washed twice with cold PBS and resuspended in 1X Binding Buffer at a concentration of 1 × 10^6^ cells/mL. Then, 100 μl of the solution (1 × 10^5^ cells) were transferred to a flow cytometer tube followed by the addition of five μl FITC Annexin-V and five μl Propidium iodide (PI). The cells were gently agitated and incubated for 15 min at room temperature in the dark. Subsequently, 400 μl of 1X Binding Buffer was added to each tube. The stained cells were analysed using BD FACS Canto II flow cytometer (BD, USA) within one hour. The BD FACSDiva version 6.1.2 software (BD, USA) was used to analyse the data.

### Detection of Caspases Activity using Colorimetric Assay

The enzymatic activity of caspase-1, -3, -8 and -9 (R&D System, USA) class of proteases in the apoptotic cells was carried out using colorimetric reaction as described by the manufacturer. Briefly, the uninfected and infected cells were detached with a scraper and the detached cells were harvested by centrifugation at 125 × g for 5 min at room temperature. The supernatant was discarded while the cell pellet (2 × 10^6^ cells) was lysed with the addition of 50 μl Lysis Buffer. The cell lysate was incubated on ice for 10 min and then centrifuged at 10,000 × g for 1 min. The supernatant was transferred into a new tube and kept on ice. The protein concentration of the cell lysate was estimated using NanoDrop UV-Visible spectrophotometers. The enzymatic reaction for caspase activity was carried out into a 96 well flat bottom microplate that measured using a microplate reader. One ml of 2X Reaction Buffer was freshly prepared by adding 10 μl of fresh DTT stock. Then, 50 μl of 2X Reaction Buffer was added to 50 μl of the cell lysate. Five μl of the caspase colorimetric substrate was added to each well. The plate was incubated at 37°C for 1 h and measured using a microplate reader at a wavelength of 405 nm. The result was expressed as fold increase in caspase activity of apoptotic cells over that of non-induced cells.

### Detection of p53 Protein Expression using a Flow Cytometer

The apoptotic protein such as p53 (Cell Signalling Technology, USA) was analysed by flow cytometer. Briefly, the uninfected and infected cells were detached with a scraper and the detached cells were harvested by centrifugation at 125 × g for 5 min at room temperature. Supernatant was discarded and the cells were washed twice with 1X PBS by centrifugation at 125 × g for five minutes. Cells were fixed with iced-cold 70% ethanol for less than one hour at 4°C and subsequently the cells were washed twice in PBS. After that, cells were incubated in blocking buffer (2% bovine serum albumin) for ten minutes and later, the cells were washed again in PBS. The cells were resuspended in PBS and subsequently, 5 × 10^5^ cells were aliquot into a new tube. Then, the cells were incubated with 20 μl of conjugated primary antibody for 1 h at room temperature and washed twice in PBS. For the unconjugated primary antibody, the cells were incubated with 5 μl goat anti-mouse IgG-PE for 30 min at room temperature. The cells were washed again in PBS and then resuspended with 0.5 ml PBS. The stained cells were analysed by BD FACS Canto II flow cytometer within 1 h. BD FACSDiva version 6.1.2 software was used to analyse the data.

### Detection of Bcl-2 and Bax Protein Expression using Western Blot

Briefly, uninfected and infected cells were detached with a scraper and the detached cells were harvested by centrifugation at 125 × g for 5 min at room temperature. After discarding the supernatant, the cell pellet (2 × 10^6^ cells) was lysed by the addition of 100 μl RIPA buffer (Nacalai Tesque, Japan) and incubated overnight at 4°C. On the next day, the supernatant was collected using centrifugation at 1650 × g for 20 min and kept at –80°C. The protein concentration of the cell lysate was estimated using the NanoDrop UV-Visible spectrophotometers. Approximately 120 μg of total protein per sample was mixed with sample loading buffer and heated for 5 min at 95°C. Acrylamide gel (10%) stacking gel (5%) was used to separate proteins with molecular weight of 15 to 100 kDa. After the sample loading, the gels were electrophoresed with the running buffer at 90 V for 2 h. The resolved protein samples from SDS-PAGE were transferred onto a polyvinylidene difluoride (PVDF) membrane using electroblotting. After assembling the gel and the sandwich membrane, the semi-dry transfer was performed with Towbin transfer buffer for 3 h at 15 V. Once the electroblotting was complete, the membrane was incubated in blocking solution (1% skimmed milk in Tris-buffered saline-Tween-20 (TBST)) for one hour at room temperature and subsequently, was washed three times with TBST. Then, the membrane was incubated with diluted primary antibody for Bcl-2 and Bax (Santa Cruz Biotechnology, Europe) in blocking solution for 1 h at 37°C or overnight at 4°C. The unbound antibody was removed by washing the membrane with TBST for 30 min; the TBST buffer was replaced every 10 min. The membrane was incubated with diluted secondary antibody conjugated to horseradish peroxidase (HRP) in blocking solution for 1 h under agitation and followed by washing step with TBST for 30 min. Chemiluminescence signal was visualised by chemiluminescence documentation system using the enhanced chemiluminescence (ECL) Western blot detection reagent (Nacalai Tesque, Japan), as described by the manufacturer. An antibody specific to β-actin (Sigma, USA) was used as a control. The intensity density value (IDV) of the targeted protein expression bands and β–actin bands was quantified by Image J 1.42.

### Statistical Analysis

Statistical analyses were performed using the Statistical Package of Sosial Sciences (SPSS) software, version 20. The data were obtained from three independent experiments (*n* = 3) and presented as the means ± standard error of the mean (SEM). Then, the data were analysed by repeated measures analysis of variance (RM ANOVA). A *p*-value of < 0.05 was considered statistically significant.

## RESULT

### Viability of Mouse Macrophage Cell Line J774A.1 in Response to BCG and BCG-MSP1C Cells

**Figure 1:**
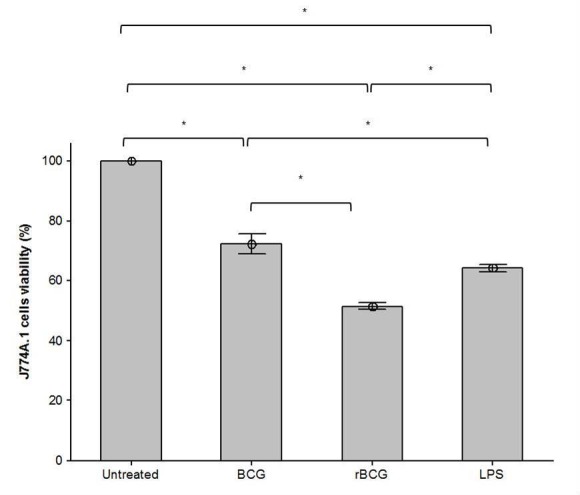
The viability of mouse macrophage cell line J774A.1 infected with BCG or BCG-MSP1C clones for 48 h. The untreated cells and the LPS-stimulated cells were used as a negative control and positive control respectively. The data are expressed as the mean of J774A.1 cell viability ± SEM of three independent experiments. The data were analysed using RM ANOVA and *p < 0.05 was considered as significantly different.

MTT assay was carried out to determine the viability of the mouse macrophage cell line J774A.1 in response to LPS, BCG or BCG-MSP1C cells at 48 h of incubation times. The viability of mouse macrophage cell line J774A.1 was significantly reduced when stimulated with LPS as well as infected with BCG and BCG-MSP1C cells in comparison with the untreated cells (100.00 ± 0.00%). However, amongst these cells, the BCG-MSP1C-infected cells showed the highest reduction in cell viability (51.63 ± 0.60%) followed by LPS-stimulated cells (64.24 ± 0.58%) and BCG-infected cells (72.45 ± 1.70%) (Fig. 1).

### Nuclear Staining of the Apoptotic Cells in Mouse Macrophage Cell Line J774A.1 in Response to BCG and BCG-MSP1C Cells

The apoptotic cells in infected mouse macrophage cell line J774A.1 were detected by the nuclear staining with Hoechst 33342. All mouse macrophage cell line J774A.1, either stimulated with LPS or infected with the BCG and BCG-MSP1C cells for 48 hours exhibited an enhanced fluorescence compared to the untreated cells (Fig. 2(A)). The enhanced fluorescence represents the condensed chromatin of apoptotic cells. The apoptotic cells were counted with at least 200 cells in ten random fields and the results were summarised in Fig. 2(B). In comparison with the untreated cells (13.93 ± 0.03%), apoptotic cells in the mouse macrophage cell line J774A.1 were significantly increased after LPS stimulation or BCG and BCG-MSP1C infection. Nevertheless, the highest percentage of apoptotic cells can be observed in the BCG-MSP1C-infected cells (62.73 ± 0.38%) compared to the LPS-stimulated cells (36.03 ± 0.55%) and the BCG-infected cells (29.87 ± 0.09%).

### Flow Cytometric Analysis of the Apoptotic Cells in Mouse Macrophage Cell Line J774A.1 in Response to BCG and BCG-MSP1C Cells

Apoptosis activity in mouse macrophage cell line J774A.1 in response to LPS, BCG or BCG-MSP1C cells for 48 h was then confirmed by the flow cytometric analysis using Annexin-V and PI stain. The cell populations were categorised into viable cells (AV^−^/PI), early apoptotic cells (AV+/PI^−^), late apoptotic cells (AV^+^/PI^+^) and necrotic cells (AV^−^/PI^+^) (Fig. 3(A)). The untreated cells comprise of 96.67% of viable cells, 4.23% of early apoptotic cells, 0.04% of late apoptotic cells and 0.05% necrotic cells. The addition of LPS into the macrophage decreased the percentage of viable cells (89.79%) but increased the percentage of early apoptotic cells (9.19%), late apoptotic cells (0.58%) and necrotic cells (0.77%) in comparison to the untreated cells. Apart from that, there was a reduction in the percentage of viable cells in the mouse macrophage cell line J774A.1 after the BCG infection (91.53%) and BCG-MSP1C cells infection (88.65%) in comparison with the untreated cells. However, the percentage of early apoptotic cells in the BCG-infected cells (6.37%) and BCG-MSP1C-infected cells (11.26%) was greater than untreated cells. The percentage of early apoptotic cells was further compared according to the type of infection. The percentage of early apoptotic in the infected cells was significantly increased when stimulated with LPS as well as infected with the BCG and BCG-MSP1C cells in comparison with the untreated cells (4.23 ± 0.39%). However, the BCG-MSP1C cells stimulated the highest of early apoptotic cells (11.26 ± 0.16%) compared to LPS (9.19 ± 0.21%) and BCG (6.37 ± 0.60%) (Fig. 3(B)).

### Caspases Activity in Mouse Macrophage Cell Line J774A.1 in Response to BCG and BCG-MSP1C Cells

**Figure 4:**
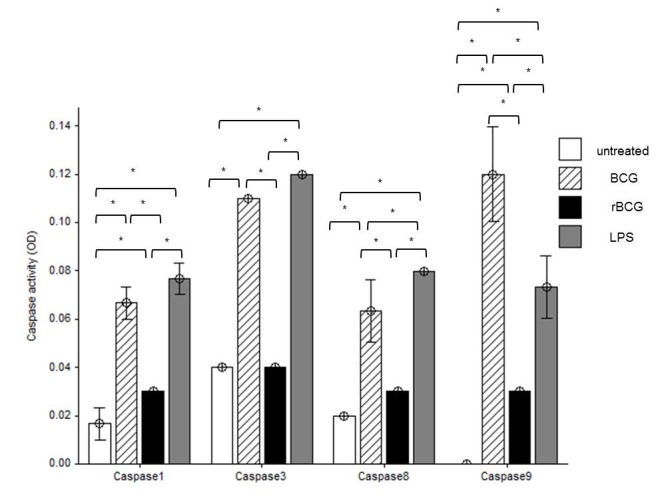
The activity of caspase 1, 3, 8 and 9 in mouse macrophage cell line J774A.1 infected with BCG or BCG-MSP1C clones for 48 hours. The untreated cells and LPS-stimulated cells were used as a negative control and positive control respectively. The data are expressed as the mean absorbance of the activity of caspase ± SEM of three independent experiments. The data were analysed using RM ANOVA and *p < 0.05 was considered as significantly different.

The colorimetric assay was carried out to determine the activity of caspases in mouse macrophage cell line J774A.1 after stimulated with LPS or infected with BCG and BCG-MSP1C cells for 48 h. Overall, the results revealed that the LPS-stimulated cells, BCG-infected cells and BCG-MSP1C-infected cells produced higher caspases activities in comparison with the untreated cells. The caspase-1, -3, -8 and -9 activities in the BCG-infected cells and LPS-stimulated cells were significantly higher than the untreated cells as well as the BCG-MSP1C infected cells (Fig. 4). However, the BCG-MSP1C-infected cells only stimulated higher activity of caspase-1 and -9 compared to untreated cells. For caspase-1 activity, the LPS-stimulated cells showed the highest activity (0.077 ± 0.003) followed by the BCG-infected cells (0.067 ± 0.003) and the BCG-MSP1C-infected cells (0.030 ± 0.000). Meanwhile, caspase-3 activity was significantly higher in the LPS-stimulated cells (0.120 ± 0.000) and BCG-infected cells (0.110 ± 0.000) in comparison with the BCG-MSP1C-infected cells (0.040 ± 0.000). No significant difference was observed in activity of caspase-3 between the rBCG-infected cells and the untreated cells. The highest caspase-8 activity was detected in the mouse macrophage cell line J774A.1 stimulated with LPS (0.080 ± 0.000) and followed by BCG infection (0.063 ± 0.007). However, caspase-8 activity in the BCG-MSP1C-infected cells was not significantly different (0.030 ± 0.000) with the untreated cells. Additionally, the BCG clone showed the highest level of caspase-9 activity in the infected cells (0.120 ± 0.010) followed by LPS (0.073 ± 0.007) and BCG-MSP1C cells (0.030 ± 0.000).

### Expression of p53 Protein in Mouse Macrophage Cell Line J774A.1 in Response to BCG and BCG-MSP1C Cells

**Figure 5:**
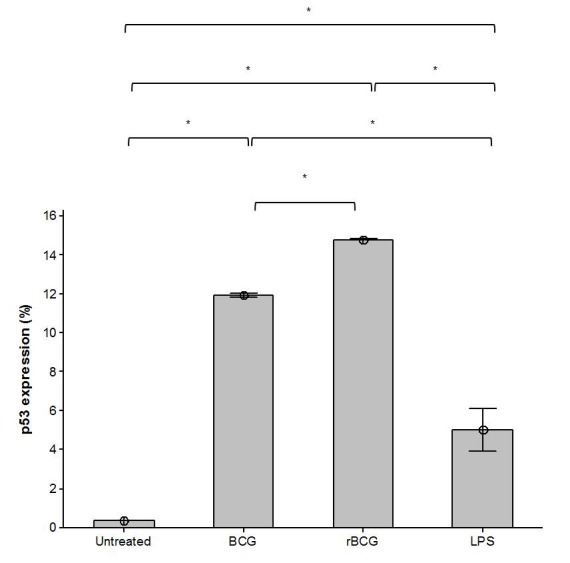
The expression of p53 protein in mouse macrophage cell line J774A.1 infected with BCG or BCG-MSP1C clones for 48 h. The untreated cells and LPS-stimulated cells were used as a negative control and positive control respectively. The data are expressed as the mean of p53 protein expression ± SEM of three independent experiments. The data were analysed using RM ANOVA and *p < 0.05 was considered as significantly different.

p53 is a tumor suppressor protein which capable of promoting apoptosis activity and ensuring that the cell death program occurs efficiently. The expression of p53 protein in the infected mouse macrophage cell line J774A.1 was determined using flow cytometric analysis. The result obtained showed that, there was a significant induction of the expression of p53 protein when the cells stimulated with LPS as well as infected with the BCG and BCG-MSP1C cells at 48 h incubation times compared to the untreated cells (0.36 ± 0.00%). However, the BCG-MSP1C-infected cells expressed the highest level of p53 protein (14.79 ± 0.01%) in comparison with the BCG-infected cells (11.93 ± 0.05%) and LPS-stimulated cells (5.02 ± 0.56%) (Fig. 5).

### Expression of Bcl-2 and Bax Proteins in Mouse Macrophage Cell Line J774A.1 in Response to BCG and BCG-MSP1C Cells

**Figure 6:**
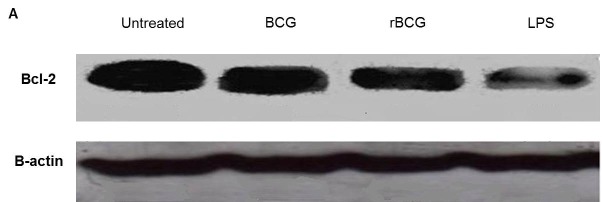

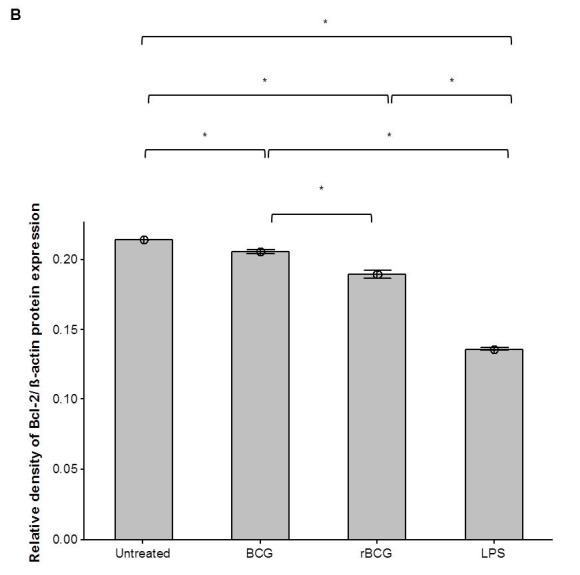
(A) Representative result of Bcl-2 and β–actin protein expression; (B) The relative density of Bcl-2/β-actin protein expression in mouse macrophage cell line J774A.1 infected with BCG or BCG-MSP1C clones for 48 h. The untreated cells and LPS-stimulated cells were used as a negative control and positive control respectively. The data are expressed as the mean relative density of Bcl-2/β-actin protein expression ± SEM of three independent experiments. The data were analysed using RM ANOVA and *p < 0.05 was considered as significantly different.

Bcl-2 is an important anti-apoptotic protein, which inhibits apoptosis. The expression of Bcl-2 protein in the mouse macrophage cell line J774A.1 after stimulated with LPS or infected with the BCG and BCG-MSP1C cells for 48 h was determined by Western Blot. A band at approximately 26 kDa was observed in the untreated cells, BCG-infected cells, BCG-MSP1C-infected cells as well as LPS-stimulated cells when the membrane was incubated with anti-Bcl-2 antibody (Fig. 6(A)). Antibody against β-actin was used as an internal control.

The density of Bcl-2 expression was then compared with the density of β-actin. The result showed that the relative density of Bcl-2/β-actin protein expression was significantly reduced when the cells stimulated with LPS or infected with the BCG and BCG-MSP1C cells in comparison with the untreated cells (0.214 ± 0.000). Among these cells, LPS-stimulated cells exhibited the highest reduction in Bcl-2 expression (0.136 ± 0.001) followed by BCG-MSP1C-infected cells (0.189 ± 0.002) and BCG-infected cells (0.206 ± 0.001) (Fig. 6(B)).

**Figure 7:**
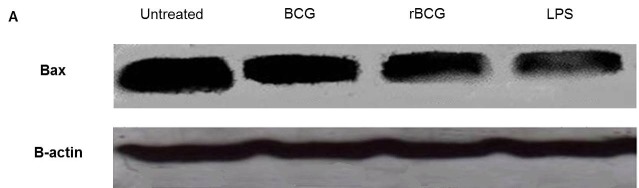

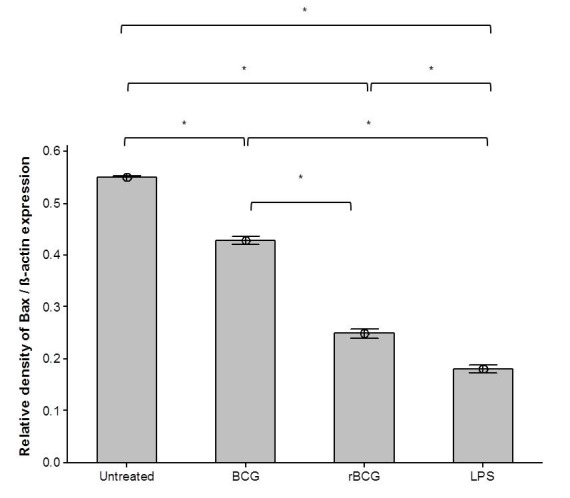
(A) Representative result of Bax and β-actin protein expression; (B) The relative density of Bax/β-actin protein expression in mouse macrophage cell line J774A.1 infected with BCG or BCG-MSP1C clones for 48 h. The untreated cells and LPS-stimulated cells were used as a negative control and positive control respectively. The data are expressed as the mean relative density of Bax/β-actin protein expression ± SEM of three independent experiments. The data were analysed using RM ANOVA and *p < 0.05 was considered as significantly different.

Bax is a member of the Bcl-2 protein family and functions as apoptosis activator. The expression of Bax protein in mouse macrophage cell line J774A.1 after stimulated with LPS or infected with BCG and BCG-MSP1C cells for 48 h was determined using Western Blot. A band at approximately 23 kDa was observed in the untreated cells, BCG-infected cells, BCG-MSP1C-infected cells as well as LPS-stimulated cells when the membrane was incubated with anti-Bax antibody. Antibody against β-actin was used as an internal control (Fig. 7(A)).

The density of Bcl-2 expression was then compared with the density of β-actin. The result showed that the relative density of Bax/β-actin protein expression in the infected cells was significantly reduced when stimulated with LPS as well as infected with BCG and BCG-MSP1C cells in comparison with the untreated cells (0.551 ± 0.000). However, LPS expressed the highest reduction of the relative density of Bax/β-actin protein expression in the infected cells (0.182 ± 0.004) followed by BCG-MSP1C cells (0.249 ± 0.005) and BCG clone (0.428 ± 0.004) (Fig. 7(B)).

## DISCUSSION

Apoptosis is the major cell death pathway which clears unwanted and harmful cells in a clean or silent manner during embryonic development, tissue homeostasis and immune regulation. The apoptotic cells can be characterised by a set of biochemical and physical changes in the nucleus, cytoplasm and plasma membrane. This pathway is essential in the prevention of local inflammatory reactions and tissue damage (Schwartzman & Cidlowski 1993; Taylor *et al*. 2008; Aguiló *et al*. 2014). During mycobacterial infection, macrophage apoptosis exerts an innate immune defense mechanism against mycobacteria (Raja 2004). The extensive apoptosis activity of the infected macrophage provides a robust protective effect in eliminating intracellular infection including malaria parasites (Keane *et al*. 2000). Elucidating the activation of the intracellular signaling pathway during a mycobacterial infection will provide great insights into the key immunological processes induced by the mycobacteria and assist in the rational design of more effective vaccines (Jo *et al*. 2007).

An earlier study revealed that our BCG-MSP1C vaccine is capable of enhancing appropriate humoral and cellular immune responses in mice. Sera of the immunised mice contained significant levels of IgG2a subclass against the MSP1C. The sera also reactived with fixed *P. falciparum* merozoites as demonstrated by indirect immunofluorescence assay (IFA) and inhibited merozoite invasion of erythrocytes *in vitro* (Nurul & Norazmi 2011). Additionally, the vaccine candidate is able to stimulate phagocytosis activity, expression of toll-like receptors (TLRs) and production of pro-inflammatory cytokines much higher than those produced by the parent BCG cells (Rapeah *et al*. 2010). The BCG and BCG-MSP1C cells are capable of reducing the viability of the infected macrophage. It is suggested that the capability of the BCG-MSP1C cells to reduce the viability of the infected cells might be associated with the apoptosis signaling pathway (Rapeah *et al*. 2010). Therefore, the present study was performed to determine the apoptosis activity and the mitogen-activated protein kinase (MAPK) expression in the mouse macrophage cell line J774A.1 infected with the BCG and BCG-MSP1C cells.

The characteristics of the apoptotic cells include the preservation of the plasma membrane’s integrity, chromatin condensation, nuclear fragmentation, cell shrinkage and formation of apoptotic bodies (Cohen 1993; Martin *et al*. 1995). In this study, the fluorescence microscopy of Hoechst-stained was used as a marker of apoptosis since it stains the condensed chromatin of apoptotic cells brighter than the chromatin of normal cells (Suzuki *et al*. 2004). The nuclear staining with Hoechst stain showed that the apoptotic cells were detected in the BCG-infected cells and BCG-MSP1C - infected cells. This finding indicates that the BCG and BCG-MSP1C cells are capable of inducing classical apoptosis activity in the infected cells.

The early apoptosis activity in the infected cells was further confirmed by detecting the binding of Annexin-V to phosphatidylserine (PS) using a flow cytometer. At the earlier stage of the apoptosis, most mammalian cell types translocate PS from the inner face of the plasma membrane to the cell surface which mediates macrophage recognition of the apoptotic cells (Fadok *et al*. 1992; Zhang *et al*. 1997). The result confirmed that the BCG and BCG-MSP1C cells stimulated a high percentage of early apoptotic activity in the infected cells compared to the untreated cells. The finding is in line with the previous study showing that infection with attenuated mycobacterial, such as *Mycobacterium bovis* BCG stimulates apoptosis in the mouse macrophage and induces direct killing of the intracellular mycobacteria (Molloy *et al*. 1994; Keane *et al*. 2000; Carrie & Hardy 2003; Sly *et al*. 2003; Chen *et al*. 2006; Krzyzowska *et al*. 2007). This result provides an early marker for a downstream study of the apoptotic pathways (Zhang *et al*. 1997).

Based on the nuclear changes and PS translocation, this study revealed that the BCG-MSP1C-infected cells exhibit stronger apoptosis effects compared to the parent BCG-infected cells. This finding was supported by the previous study which reported that the recombinant tuberculosis vaccine induces apoptotic vesicles (Farinacci *et al*. 2012). The difference of the apoptotic response between the BCG and BCG-MSP1C-infected cells supports our hypothesis that the BCG-MSP1C is proficient to exert the best modulator role of apoptosis, contributing to a very potent innate immune response in comparison to the BCG. We hypothesized that the presence of MSP-1C in the BCG renders the BCG-MSP1C to induce apoptosis activity in the infected cells. However, the actual mechanism by which the BCG-MSP1C promotes the apoptotic response in the infected cells still remains unknown and requires more study.

There was evidence showing that the action of specific proteins belonging to the family of Bcl-2 proteins influences the apoptotic response in the infected macrophage (Krzyzowska *et al*. 2007). The capability of the clones to stimulate the expression of the pro and anti-apoptotic protein was further evaluated. In this experiment, the expression of an anti-apoptotic protein Bcl-2 which inhibits the apoptosis was reduced in the BCG-infected cells and BCG-MSP1C-infected cells in comparison with the untreated cells. The result is consistent with a previous study reporting that the anti-apoptotic protein Bcl-2 was down-regulated in the human mononuclear phagocytes after an infection with *Mycobacterium bovis* BCG (Klingler *et al*. 1997). Moreover, the BCG-MSP1C-infected cells had a lower level of the anti-apoptotic protein Bcl-2 than the parent BCG-infected macrophage, indicating that the BCG-MSP1C-infected cells suppressed the actions of the anti-apoptotic activity of the Bcl-2 protein resulted in the induction of the apoptosis activity in the infected macrophage.

An earlier study reported that the down-regulation of Bcl-2 was accompanied by an upregulation of Bax in order to promote apoptosis (Decaudin *et al*. 1997). Conversely, the decreased expression of Bax was observed in BCG-MSP1Cinfected cells compared with the BCG-infected cells, indicating that the Bax is not an apoptotic mediator during the infection. Similar results were observed in a previous study reporting there was no change in the Bax protein expression when mononuclear phagocytes were induced by the mycobacteria (Klingler *et al*. 1997).

p53 is a tumor suppressor protein which interacts with the multi domain members of the Bcl-2 family to induce mitochondrial outer membrane permeabilization (MOMP) leading to the macrophage apoptosis (Miyashita *et al*. 1994; Vaseva & Moll 2009). Notably, the cells infected with the BCG and BCGMSP1C cells resulted in a marked increase of p53 expression compared to the untreated cells. The ability of the BCG clones to activate the expression of the p53 protein in the macrophage is well evidenced in the previous studies. In the present study, we found that the expression of p53 protein in the BCG-MSP1C-infected cells was up-regulated compared to those in the BCG-infected cells. It showed that the p53 dependant pathway is one of the possible mechanisms to promote apoptosis in the infected cells. A similar finding was also observed in a study by Danelishvili *et al*. (2003) who demonstrated that the p53 protein was induced after a *Mycobacterium tuberculosis* H37Rv infection of the human macrophages.

In order to elucidate the apoptotic pathways in the infected cells following BCG and BCG-MSP1C infections, caspase-1, -3, -8 and 9 activity were measured. It is known that the morphological alterations of apoptosis are mediated by the activation of an evolutionarily conserved and unique class of intracellular proteases known as caspases (Taylor *et al*. 2008). Several studies reported that apoptosis could be initiated and executed through two main pathways: the extrinsic caspase-8 dominant cascade and the intrinsic caspase-9 dominant cascade (Moffitt *et al*. 2010).

The extrinsic (death receptor-mediated) apoptosis pathway involves the ligation and oligomerisation of the death receptor by their cognate ligands in turn to caspase cascade (Chen & Wang 2002). Death receptors, including Fas, TNFR, tumor necrosis factor-related apoptosis-inducing ligand RI (TRAIL-RI), and TRAIL-RII, initiate apoptosis in response to ligation with their respective death ligands (Ashkenazi & Dixit 1998). Signals transduced through TNFR1 (p55) can induce an activation of proteases which are recognised mediators of apoptosis by proteolytic cleavage of the “death substrates” poly (ADP-ribose) polymerase. The activation of caspase-8 may directly activate caspase-3, which results in the apoptotic cell death (Pope 2002; Labbe & Saleh 2008; Natarajan *et al*. 2011). This study demonstrated that the BCG-infected cells expressed a significant increase in caspase-8 activity in comparison with the untreated cells. It revealed that the extrinsic pathway is responsible for triggering the apoptosis in BCG-infected cells. This data is in line with the previous study reporting that activity of caspase-8 significantly increased only at day 3 in BCG-infected cells (Krzyzowska *et al.* 2008). In our experiment, caspase-8 activity in the BCG-MSP1C-infected cells was not significantly different with the untreated cells indicating the absence of caspase-8 activation in BCG-MSP1C-infected cells. This finding revealed that BCG-MSP1C cells was not capable to induce apoptosis through the extrinsic pathway.

A significant increase in caspase-9 activity in the infected cells was observed after the BCG and BCG-MSP1C infections. This finding indicates that the infected cells undergo intrinsic pathway leading to apoptosis. The previous study done by Krzyzowska *et al.* (2008) reported that BCG-induced apoptosis was capable of increasing the activity of caspase-9. The activity of caspase-9 is initiated when the apoptotic signals induce the loss of mitochondrial integrity and the release of pro-apoptotic molecules, including cytochrome c. Once in the cytosol, cytochrome c binds to the apoptotic protease activating factor-1 leading to the formation of the apoptosome. This process results in the activation of caspases 9 and 3, thus triggering apoptosis (Green & Reed 1998; Imawati *et al*. 1999; Danial & Korsmeyer 2004). Apart from that, the level of caspase-9 activity in the BCG-MSP1C-infected cells was lower than the BCG-infected cells, indicating the presence of MSP-1C in the BCG-MSP1C construct reduced the ability of the parent BCG to activate the intrinsic pathway of the apoptosis. However, the molecular events underlying this phenomenon remain unknown and require further investigation, especially on cytochrome c activity which was not determine in this study.

Caspase-3 is an important step proceeding PS translocation and apoptotic nuclear morphology (Amarante-Mendes *et al*. 1998). Moreover, caspase-3 activity is dependent upon activation by an upstream, initiator caspases such as caspase-8 and -9 in an apoptotic pathway (Thornberry & Lazebnik 1998). Our observation is in line with a previous finding by Krzyzowska *et al*. (2007) who clearly showed that the BCG was capable of increasing the activity of the caspase-3 in the infected macrophages. However, similar to the caspase-9 activity, the caspase-3 activity of the BCG-MSP1C-infected cells was significantly lower than that of the BCG-infected cells. This finding indicates the exclusion role of caspase-3 in the apoptosis induction upon the BCG-MSP1C infection. This suggests that there could be another pathway that BCG-MSP1C adopts for inducing apoptosis in the macrophage. A previous study reported that macrophage infected with *Mycobacterium bovis* undergoes caspase-independent apoptosis, which is mediated by an apoptotic inducing factor (AIF) (Lee *et al.* 2006; Vega-Manriquez *et al*. 2007). AIF is an apoptotic effector which causes chromatin condensation and deoxyribonucleic acid (DNA) fragmentation (Cregan *et al*. 2004; Cande *et al.* 2002; Donovan & Cotter 2004).

Caspase-1, formerly known as interleukin (IL)-1-converting enzyme is best established as the protease responsible for the processing of the key pro-inflammatory cytokine IL-1β from an inactive precursor to an active, secreted molecule (Kostura *et al*. 1989; Hogquist *et al*. 1991; Schlegel *et al*. 1996; Schumann *et al*. 1998). Caspase-1 is an inflammatory caspase which induce the production of IL-1β (Labbe & Saleh 2008). A previous study demonstrated the ability of our BCG-MSP1C cells to enhance the production of the IL-1β in infected macrophage (Rapeah *et al*. 2010). Therefore, the activity of caspase-1 in the infected cells was determined in this study, to evaluate the involvement of caspase-1 activity in the cell death stimulated by the BCG-MSP1C cells. By comparing it with the untreated cells, the activity of caspase-1 was up-regulated in the BCG-infected cells and BCG-MSP1C-infected cells. This finding proves that caspase-1 plays a role in modulating the cell death during the infection. Nevertheless, the caspase-1 activity of the BCG-MSP1C-infected cells was significantly lower than BCG-infected cells, suggesting that caspase-1 may not be the sole factor involved in such cell death (Natarajan *et al*. 2011). In conclusion, all the objectives of this study were achieved. The presence of MSP-1C of *P. falciparum* in BCG increases the apoptosis activity in the infected macrophage by down-regulating the anti-apoptotic Bcl-2 protein and stimulating the expression of p53 protein. The BCG-MSP1C cells also increased the expression of caspase-1 and -9 indicating the involvement of the intrinsic (mitochondrial-mediated) apoptosis pathway during this infection. The findings are important in order to understand the mechanism of how the BCG-MSP1C cells can stimulate the elimination activity of the host macrophage during a malaria infection. However, further investigation is required to understand the actual mechanism underlying this phenomenon.

## Figures and Tables

**Figure 2(A):**
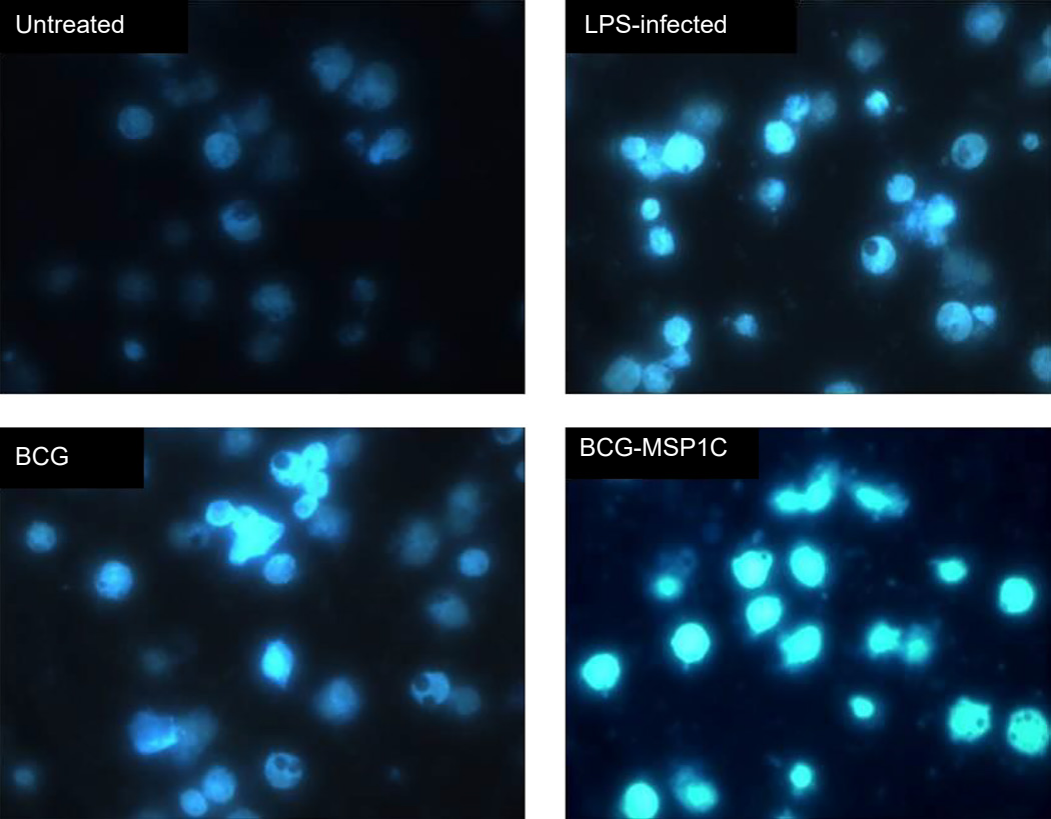
Hoechst 33342 nuclear staining of apoptotic cells in mouse macrophage cell line J774A.1 after being infected with BCG or BCG-MSP1C clones for 48 h. The untreated cells and LPS-stimulated cells were used as a negative control and positive control respectively. The stained cells were examined using fluorescence microscopy and photographed at 40× magnification.

**Figure 2(B):**
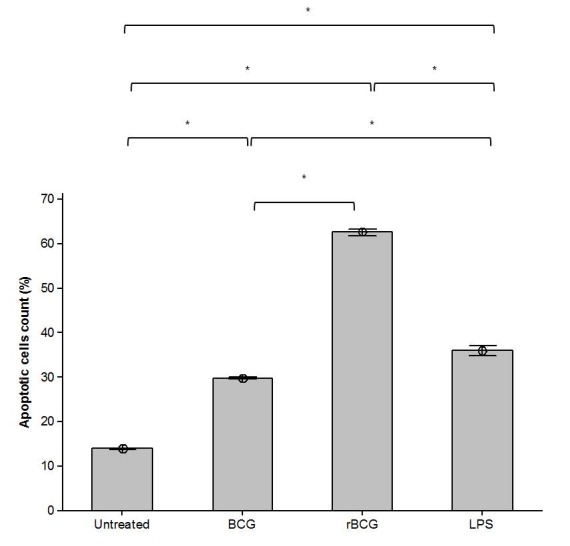
The percentage of apoptotic cells in mouse macrophage cell line J774A.1 infected with BCG or BCG-MSP1C clones for 48 h. The untreated cells and the LPS-stimulated cells were used as a negative control and positive control respectively. The cells were scored as apoptotic if they exhibited unequivocal nuclear chromatin condensation. The data are expressed as the mean percentage of apoptotic cells ± SEM of ten different fields. The data were analysed using RM ANOVA and *p < 0.05 was considered as significantly different.

**Figure 3(A):**
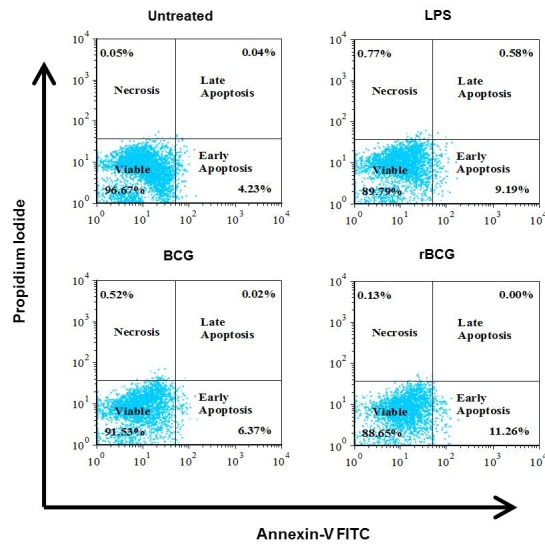
Flow cytometric analysis of apoptotic cells using Annexin-V and propidium iodide staining in mouse macrophage cell line J774A.1 infected with BCG or BCG-MSP1C clones for 48 h. The untreated cells and LPS-stimulated cells were used as a negative control and positive control respectively. The data are expressed as the mean percentage of cell population of three independent experiments. The data were analysed using RM ANOVA and *p < 0.05 was considered as significantly different.

**Figure 3(B):**
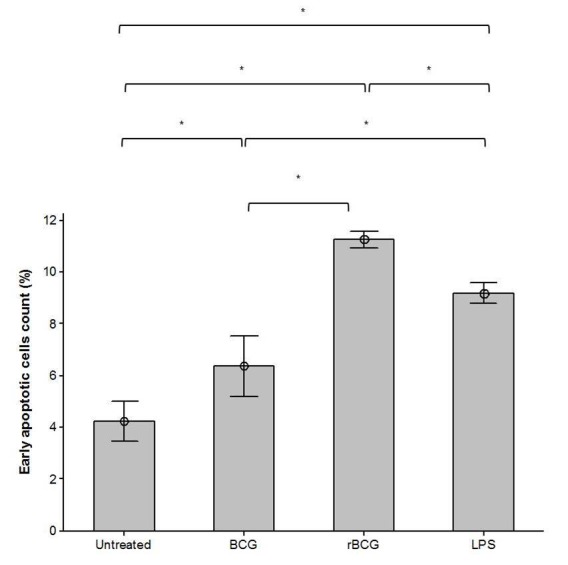
The percentage of early apoptotic cells in mouse macrophage cell line J774A.1 infected with BCG or BCG-MSP1C clones for 48 h. The untreated cells and LPS-stimulated cells were used as a negative control and positive control respectively. The data are expressed as the mean percentage of early apoptotic cells ± SEM of three independent experiments. The data were analysed using RM ANOVA and *p < 0.05 was considered as significantly different.
